# Biofabrication of Silver Nanoparticles Using *Pergularia tomentosa* Extract and Evaluation of Their Antibacterial, Antioxidant, and Cytotoxic Properties

**DOI:** 10.3390/life14121639

**Published:** 2024-12-10

**Authors:** Munirah F. Aldayel

**Affiliations:** Department of Biological Sciences, College of Science, King Faisal University, Al-Ahsa 31982, Saudi Arabia; maldayel@kfu.edu.sa

**Keywords:** silver nanoparticles, *Pergularia tomentosa*, antibacterial, antioxidant, anti-inflammatory

## Abstract

The biosynthesis of silver nanoparticles using plant extracts is a promising field of research because of the useful biomedical applications of metal nanoparticles. In this study, the antibacterial and antioxidant properties of silver nanoparticles biosynthesized with the aqueous leaf extract of *Pergularia tomentosa* were defined using a simple, eco-friendly, consistent, and cost-effective method. The leaf extract of *Pergularia tomentosa* (PT) served as a capping and reducing agent to biosynthesize silver nanoparticles. The effects of several parameters, such as the concentration of AgNO_3_, ratio of AgNO_3_ to extract, pH, and incubation time, were examined to optimize the synthesis process. In total, 5 mM of AgNO_3_, a 1:0.06 ratio of AgNO_3_ to *Pergularia tomentosa* extract, pH 9.0, and reaction mixture incubation for 24 h were found to be the ideal parameters for biosynthesizing silver nanoparticles (AgNPs). UV–visible spectroscopy, X-ray diffraction (XRD), Fourier-Transform Infrared Spectroscopy (FTIR), and scanning electron microscopy were used to characterize the biosynthesized *Pergularia tomentosa* silver nanoparticles (PT-AgNPs). Gram-positive bacteria (*Staphylococcus aureus* and *Bacillus cereus*) and Gram-negative bacteria (*Salmonella enteritides* and *Escherichia coli*) were used to test the PT-AgNPs’ antibacterial activity. The presence of different functional groups was determined using FTIR. The AgNPs were hexagon shaped. The nanoparticles were more toxic against *S. enteritides* than both *B. cereus* and *E. coli*. In antioxidant analyses, the AgNPs were found to be as strong at free radical scavenging as gallic acid (standard), with IC_50_ values of 0.69 and 22.30 μg/mL for DPPH and ABTS radicals, respectively. Interestingly, the PT-AgNPs displayed increased anti-inflammatory activity compared with the *P. tomentosa* leaf extract (79% vs. 59% at 500 µg/mL). The PT-AgNPs did not display any cytotoxicity against the MCF-7 cell line at the MIC. In conclusion, silver nanoparticles fortified with *Pergularia tomentosa* extract exhibited potential as effective antibacterial, anti-inflammatory, and antioxidant agents, suggesting their viability as alternatives to commercially available products.

## 1. Introduction

Extensive research is being carried out on AgNPs in several fields, including medicine, pharmacology, and healthcare [1,2]. The antioxidant, antimicrobial, and anti-inflammatory properties of AgNPs have been recognized in the literature [2,3]. Reduced silver is toxic to microbes due to its ability to destroy their cell walls and disturb their normal function and growth. It does so through interactions between Ag ions and macromolecules in microbial cells. For instance, AgNPs inhibit protein synthesis and alter cell membrane permeability, resulting in cell death [4]. The greater biological potential of AgNPs than that of Ag ions might be due to their reduced size and higher surface area-to-volume ratio.

Recently, an innovative procedure for biosynthesizing metal nanoparticles with suitable morphologies and sizes has drawn the interest of scientists in the area of nanotechnology. Nanoparticles have been biosynthesized through biological approaches due to their improved viability and compatibility and their significant environmental friendliness [4]. The use of plant extracts to biosynthesize metal nanoparticles has increased because of their distinctive physicochemical characteristics, which are related to their bulk materials and the richness of plant extracts; these extracts often contain several biologically active compounds and are safe for people and the environment [5,6,7]. Different plants have been used to biosynthesize silver nanoparticles, such as *Allium cepa* [8], *Annona squamosa* leaf and fruit extracts [9], *Curcuma longa* extract [10], Calotropis-gigantean-mediated leaf extract [11], and *Achillea millefolium* [12], which act as bioreductants for the preparation of AgNPs.

*Pergularia tomentosa* is a spontaneous xerophyte shrub from the Asclepiadaceae family and is mostly distributed across the Sahara Desert, Sinai (Egypt), the Arabian Peninsula, Afghanistan, and Pakistan. The leaves are used as a dressing to treat scorpion bites and abscesses [13,14,15] and skin diseases [16]. Its leaves are also used in traditional medicine for their laxative, depilatory, and anthelmintic properties and as poultices and abortifacients. Phytochemical examinations of different plant extracts from *P. tomentosa* have shown glycosides, saponins, alkaloids, tannins, flavonoids, anthraquinones, and volatile oils [17]. However, very few studies have described the antimicrobial, antioxidant, and anti-inflammatory effects of *P. tomentosa* [18,19,20,21,22]. This is the first report to explore a method for the biosynthesis of AgNPs using an extract of *P. tomentosa* as a reducing and capping agent and to evaluate the biological activities of the resulting nanoparticles.

In the current study, we developed a simple approach to biofabricate AgNPs from *P. tomentosa*. The antioxidant, antibacterial, cytotoxic, and anti-inflammatory potentials of the biosynthesized AgNPs were examined to evaluate their pharmaceutical and biomedical significance.

## 2. Materials and Methods

### 2.1. Plant Collection

The *Pergularia tomentosa* plant was harvested in the Al-Ahsa-Dammam Road district of eastern Saudi Arabia at an altitude of 2515 m, 28′45″ N latitude, and 83′23″ E longitude. Its various parts, including leaves, fruits, and roots, were collected in their wild and natural habitat. The plant was identified by the local people as a medicinally important plant. Collection was performed in April 2021. Afterward, its herbarium was prepared, and a taxonomist at the Cairo University Herbarium and Plant Laboratories, Egypt, identified it as *Pergularia tomentosa* of the family Asclepiadaceae with voucher code no. TD-XX2 (LATD). Fresh leaves were separated and further processed to prepare the leaf extract.

### 2.2. Extract Preparation

Fresh leaves were washed with tap water and then rinsed with deionized water. The leaves were then dried for 3 days and crushed into powder using an electric blender. In total, 5 g of leaf powder was suspended in a beaker of distilled water (250 mL) and heated in a water bath at 75–80 °C for 25 min. The remaining extracts were filtered in a conical flask, cooled down, and refrigerated at 4 °C for further use in AgNP synthesis. AgNO_3_ (25 mM, 1 M) solution was made by mixing AgNO_3_ flakes in deionized water. This solution was used to biosynthesize AgNPs to test their antibacterial and antioxidant activities.

### 2.3. Preliminary Phytochemical Screening

Secondary metabolite examination of the aqueous extract of *P. tomentosa* was performed to confirm the presence or lack of potential biologically active secondary metabolites, namely tannins, saponins, flavonoids, alkaloids, and polyphenols. Conventional biochemical analysis approaches were used. Standard biochemical protocols were performed following the procedures in [23].

### 2.4. Gas Chromatography-Mass Spectroscopy (GC-MS) Analysis

Bioactive components in *P. tomentosa* leaves were identified via GC-MS using an Agilent 7890B instrument (Agilent Technologies, Inc., Santa Clara, CA, USA) equipped with Mass Hunter acquisition software, version 10. The instrument featured a highly inert HP-5MS capillary column with a nonpolar phase, measuring 30 m in length and 0.25 mm in internal diameter, which was coated with a 0.25 µm film. Helium was used as the carrier gas, flowing at a rate of 1 mL/min. The sample injector was set at a temperature of 250 °C. The temperature was maintained at 50 °C for 5 min, followed by a gradual increase to 250 °C at a rate of 100 °C per minute. Phytochemicals were identified by comparing the developed spectra with the data available in the NIST library. The percentage peak area for each compound was calculated relative to the total peak area in the chromatogram [24].

### 2.5. Biosynthesis of AgNPs

The AgNO_3_ solution (25 mM, 1 M) was prepared by mixing AgNO_3_ flakes in deionized water, followed by AgNO_3_ and leaf extracts at a ratio of 1:10 *v*/*v*, to produce a 50 mL volume. The mixtures were incubated at room temperature until the yellow color of the solution turned dark brown. The samples were then centrifuged at 5000 rpm for 30 min, and the supernatant was discarded. Deionized water (5 mL) was added to the precipitate and centrifuged again under the same conditions. The final precipitate was placed in a hot-air oven for 25 min at 65 °C, and the dried form was used in further experiments [25,26].

### 2.6. Optimizing the Green Synthesis of AgNPs

Different concentrations of silver nitrate (1.0–5.0 mM) were mixed with *Pergularia tomentosa* leaf extract in various ratios (1.0:0.005–1.0:0.06 (*v*/*v*)) under constant stirring in laminar airflow. To optimize pH for PT-AgNP biosynthesis, the pH of the solution was changed from 4 to 9 using 0.1 N of HCl and 0.1 N of KOH. Additionally, the effect of the incubation period was examined by incubating the reaction mixture for times from 20 min to 24 h. The PT-AgNPs were biosynthesized by reducing silver ions using different components of *Pergularia tomentosa* in all optimization tests. This was observed visually and by recording the UV–vis spectra of the solutions. After sonication for 20 min, the biosynthesized PT-AgNPs were centrifuged at 25,000 rpm for 15 min. The supernatant was discarded to collect the PT-AgNP pellet, which was washed 3 times with distilled water and dried overnight in an oven at 65 °C.

### 2.7. Characterization of Silver Nanoparticles

A spectrophotometer (Shimadzu UV-1601, Tokyo, Japan) was used to obtain the ultraviolet spectral data. A correlation between absorption concentration and intensity was shown using the spectral data. After completing the reaction, 1 mL of the suspension was taken from the purified samples (*P. tomentosa* and PT-AgNPs) and sonicated at 6000 rpm for 10 min. UV–vis spectra were obtained between 200 and 800 nm at 1 nm intervals. For FTIR, aqueous extracts of *P. tomentosa* and PT-AgNPs were used. In total, 10 mg of the sample was encased in a KBr pellet (100 mg) to create translucent sample discs. Next, FTIR spectroscopy was performed on the sample material using a Perkin-Elmer Spectrophotometer (Shelton, CT, USA). The Z average size (hydrodynamic diameter), size distribution (polydispersity index, PDI), and zeta potential (surface charge) were analyzed using a zeta sizer (Malvern Instruments, Model 3000 HSA, Malvern, UK), and the results were obtained using Malvern ZS Nano software, version 3.30. The crystallinity was measured using an XRD machine enhanced with Cu (copper) Ka radiation (1.54187 nm wavelength). The formation, structure, and composition of the AgNPs were tested using a Ni (nickel) filter under 40 kV/20 mA operation conditions. Then, PT-AgNPs biosynthesized under optimum conditions were characterized via scanning electron microscopy (SEM, JEOL 6460LV, Tokyo, Japan), high-resolution transmission electron microscopy (HRTEM, Tecnai G2 F20, FEI, New York, NY, USA), energy dispersion X-ray spectroscopy (EDX), elemental mapping, and selected area diffraction (SAED) patterns.

### 2.8. Antibacterial Tests

The antibacterial action of the PT-AgNPs was examined against different bacterial strains, namely, *Bacillus cereus* ATCC 10876, *Staphylococcus aureus* ATCC 32571, *Salmonella enteritidis* ATCC 14028, and *Escherichia coli* RIMD 05091078. The bacteria used in this study were clinical isolates obtained from Medicine College, King Faisal University, Al-Ahsa, Kingdom of Saudi Arabia.

### 2.9. Antibacterial Action

#### 2.9.1. Kirby–Bauer Disk Diffusion Assay

The antimicrobial activities of the PT-AgNPs and plant extract were tested against *S. aureus*, *S. enteritidis*, *E. coli*, and *B. cereus* and compared with antibiotic (chloramphenicol, positive control) and DMSO (negative control) using an agar disk diffusion assay. Briefly, after plating each strain on a sterile Mueller–Hinton agar plate (Oxoid, Italy), antibiotics and virgin disks were placed on the plates. In detail, sterile disks were loaded with 20 μL of silver nanoparticles, plant extract, DMSO, and antibiotics. All experiments were performed in aseptic conditions in a laminar airflow cabinet, and two replicates were used for each strain. The plates were incubated aerobically at 37 °C for 24 h. The zones of inhibition for the AgNPs and antibiotics were measured and expressed in millimeters (zone of inhibition ± SD) [27].

#### 2.9.2. Minimum Inhibitory Concentration

The minimum inhibitory concentration (MIC) was determined using the microdilution assay, according to Clinical and Laboratory Standards Institute (CLSI) guidelines (CLSI, 2017). Briefly, *S. enteritidis* bacteria were grown on Brain Heart Infusion agar (BHI, Scharlau, Milan, Italy), and three or four colonies were suspended in fresh sterile saline solution to reach an initial concentration of 1.5 × 10^7^ CFU/mL. In total, 100 µL of the 1:100 diluted cell suspension was dispensed into each well in a 96-well microtiter plate. *S. enteritidis* bacteria were exposed to a twofold dilution series of antibacterial agents in concentrations ranging from 512 to 1 µg/mL [28].

### 2.10. Time–Kill Test for AgNPs

The time–kill test offers an improved understanding of the kinetics of bacterial growth and bactericidal activity when treated with PT-AgNPs, helping to define the optimum concentration and exposure time necessary to kill bacterial pathogens efficiently. Log10 CFU/mL facilitates an easier explanation and assessment of bacterial concentrations, as it converts the data to a logarithmic scale. The time–kill assay was carried out on test bacteria using several concentrations of PT-AgNPs. The PT-AgNPs were diluted in MHB medium containing the bacterial inoculum to obtain concentrations of 0 × MIC, 0.5 × MIC, 1 × MIC, 2 × MIC, and 4 × MIC for the bacterial species. At specific time intervals (0, 0.5, 1, 2, 4, and 6 h), 10 μL samples were diluted with 1% sterile phosphate buffer saline and spread on MHA plates. These plates were incubated at 37 °C for 24 h. The total plate count (TPC) was then expressed as Log10 CFU/mL and plotted against time.

### 2.11. Effect of AgNPs on the Leakage of Sugars and Proteins Through the Membrane

The turbidity of the fresh bacterial cultures (according to antimicrobial screening and MIC_50_ tests, only the susceptible ones) was adjusted to a 0.5 McFarland standard. They were then inoculated at 2% (*v*/*v*) in Nutrient Broth (NB) and incubated overnight at 30 °C. Then, cultures were centrifuged at 4000 rpm for 20 min at 4 °C. Supernatants were discarded, and cells were treated with the MIC of PT-AgNPs, mixed well, and incubated overnight at 30 °C. Untreated cell suspensions in physiological saline solution were used as negative controls. Then, treated and control cultures were centrifuged at 5000 rpm for 5 min. The supernatants of the pellets were used for sugar and protein leakage assays.

#### 2.11.1. Protein Leakage 

Volumes of 500 μL of bacterial supernatant and 500 μL of Coomassie Brilliant Blue (CBB-250) solution were mixed and incubated in the dark at room temperature for 5 min. The absorbance of the samples was measured at 595 nm using a microplate reader. Standard BSA (bovine serum albumin) was used for the calibration curve [29,30].

#### 2.11.2. Leakage of Total Sugar in the Medium

The bacterial suspension was added to the NB at 37 °C with shaking at 180 r/min overnight. A 1 mL suspension was added to 7 mL of NB with MIC PT-AgNPs. The mixture was incubated for 24 h. The samples were centrifuged at 10,000× *g* for 5 min. Volumes of 50 μL of bacterial supernatant and 200 μL of anthrone solution were mixed and then cooled in ice water for 5 min. Then, the mixture was water-bathed for 10 min. The absorbance was measured at 620 nm using a microplate reader [31].

### 2.12. Scanning Electron Microscopy

The morphology of the *S. enteritidis* cultured with PT-AgNPs (MIC) for 24 h was observed via scanning electron microscopy (SEM). The bacterial suspension contained 1–2 × 10^8^ CFU/mL. Briefly, stocks of PT-AgNPs diluted in MH-B were prepared at the MIC and added to the bacterial suspension. The samples were incubated at 37 °C, and after 24 h, 100 μL aliquots were centrifuged, and the pellet was washed with PBS. Each sample was then fixed with 100 μL of 4% *v*/*v* paraformaldehyde on nitrocellulose (NC) filter membranes with a 0.2 μm pore size for 15 min and dehydrated in increasing concentrations (30–100% *v*/*v*) of ethanol. Samples were finally dried using hexamethyldisilazane (HMDS), sputter-coated with a 20 nm thick layer of gold (Baltec SCD 050), and observed under a scanning electron microscope at an accelerating voltage of 15 kV (JEOL JSM-6390 LV, Peabody, MA, USA).

### 2.13. Determination of Antioxidant Activity

#### 2.13.1. Determination of the Total Antioxidant Capacity Using the DPPH Method

The biosynthesized PT-AgNPs were tested for their antioxidant activity using the DPPH method [4]. PT-AgNPs (0.2, 0.4, 0.6, 0.8, or 1 mg/mL) were mixed with a 3 mL methanolic solution containing DPPH radicals (0.1 mM). After 30 min, absorbance was determined at 517 nm. The percent inhibition of activity was calculated as follows: [(Ac − As)/Ac] × 100 (Ac = absorbance without PT-AgNPs; As = absorbance with PT-AgNPs). The results were expressed as IC_50_, the concentration of the sample required to inhibit 50% of the DPPH concentration.

#### 2.13.2. Determination of the Total Antioxidant Capacity Using the ABTS Method

Following the 2,2′-azino-bis (3-ethylbenzothiazoline-6-sulfonic acid) (ABTS) kit’s instructions, the total antioxidant capacity was found using the ABTS method. The number of samples to be examined was considered when preparing the ABTS mother liquor. After thoroughly mixing the oxidant and ABTS solutions at room temperature, they were left in the dark for 12 to 16 h. Before usage, 80% ethanol was added to the ABTS mother liquor to dilute it. To create the sample for testing, a specific concentration of the sample was combined with two milliliters of methanol, centrifuged, and diluted on a gradient. Subsequently, 10 μL of the sample was put into each well of the plate, followed by 200 μL of the ABTS working solution. The absorbance A1 was measured at a wavelength of 734 nm after 5 min of dark incubation at room temperature. The calculation methods for the DPPH radical scavenging rate and the ABTS+ scavenging rate were identical.

### 2.14. Anti-Inflammatory Activity of Biosynthesized AgNPs

#### Suppression of Protein Denaturation

Protein denaturation was suppressed to determine the anti-inflammatory action of the *P. tomentosa* extract and the biosynthesized silver nanoparticles, as previously described [29]. First, 2 mL of the leaf extract or PT-AgNPs was added to 2.5 mL of PBS and mixed with an egg solution. The combinations were heated to 70 °C after being incubated at 37 °C. The turbidity was determined with a spectrophotometer at 660 nm. The percentage of protein denaturation suppression was calculated as follows:

Percentage of suppression of protein denaturation = 100 × [1 − Absorbance (sample)/Absorbance (Control)]

### 2.15. Cytotoxicity Study

Human breast adenocarcinoma cells (MCF-7 cell line) obtained from ATCC (#CRL-2014) were cultured in high-glucose Dulbecco’s modified Eagle’s medium (DMEM; Sigma-Aldrich, MO, USA) supplemented with 10% heat-inactivated fetal bovine serum (FBS) (Sigma-Aldrich), 4 mM of L-glutamine (Sigma-Aldrich), and 1% penicillin/streptomycin (Gibco Invitrogen, Carlsbad, CA, USA). Cells were cultured at 37 °C in a 5% CO_2_ atmosphere, with the medium changed every 2 days. Isolated cells were cultured in an RPMI-1640 medium supplemented with 1% penicillin/streptomycin (P/S) and 12% FBS (Gibco Invitrogen, Carlsbad, CA, USA). Non-adherent cells were collected after 24 h via centrifugation and re-cultured in fresh medium. For the cytotoxicity assay, cells were treated with different concentrations of PT-AgNPs in 96-well plates for 48 h, and an MTT cell proliferation assay kit (Sigma-Aldrich, Chemicon^®^, Seoul, Republic of Korea) was used to measure cell viability. Cells were incubated with medium containing 0.5 mg/mL of MTT to metabolize them into formazan. The optical density was measured at 550 nm using an ELISA plate reader. Values are expressed as a percentage of the control untreated cells [30]. Morphological modifications were detected, and images were taken under an inverted light microscope (Olympus, Center Valley, PA, USA) after 48 h. The same cell spot was marked and captured.

## 3. Results

### 3.1. Phytochemical Screening of Pergularia tomentosa Leaf Extract

A phytochemical examination of the leaf extract of *P. tomentosa* showed alkaloids, flavonoids, tannins, saponins, and glycosides (Supplementary Table S1). A gas chromatography–mass spectrometric investigation showed nine main active compounds and their locations, as demonstrated by several peaks in the chromatogram (Supplementary Figure S1 and Supplementary Table S2). The major compounds were ethanol,1-(2-butoxyethoxy) (16.7%), cyclohexane propanol (11.2%), 2-methoxy6-methyl pyrazine (13.5%), 11,3-dioxane (6.6%), 2-propenoic acid octyl ester (6.21%), dihydro methyl jasmonate (7.54), 1,2-benzene dicarboxylic acid (7.22%), and 4-(bromomethyl) cyclohexane-1-ol (2.05%).

### 3.2. Biosynthesis of Silver Nanoparticles

**Figure 1:**
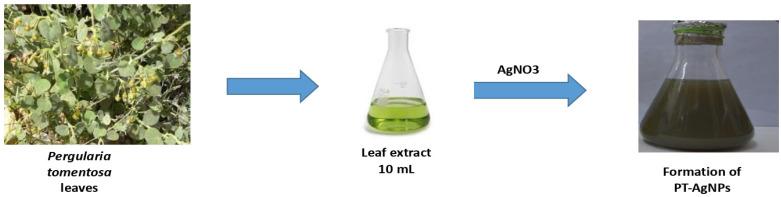
Green biosynthesis of PT-AgNPs using *Pergularia tomentosa* leaf extract. The biosynthesis of PT-AgNPs was performed by adding 5 mM of AgNO_3_ to the plant extract for 24 h under dark conditions.

Silver nanoparticles were biosynthesized from *P. tomentosa*, and we investigated the ability of the plant extract’s active components to work as bioreductants to reduce the silver ions into silver. The color change to colloidal brown after the addition of AgNO_3_ was also investigated (Figure 1).

### 3.3. Optimization of Silver Nanoparticle Synthesis

**Figure 2:**
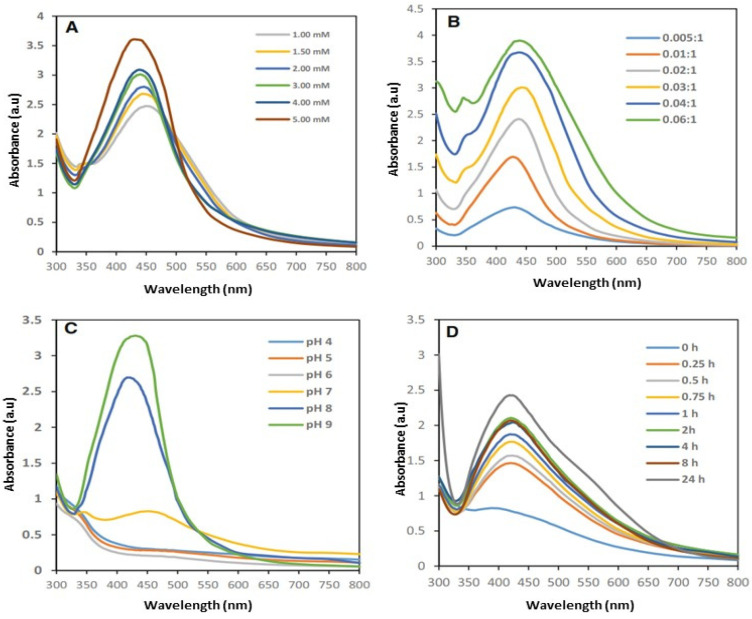
UV–visible absorption spectra of biofabricated PT-AgNPs (**A**) at different silver nitrate concentrations; (**B**) at different ratios of *Pergularia tomentosa* leaf extract to silver nitrate; (**C**) at different pH values; (**D**) at different incubation periods.

Several reaction parameters were examined for the optimum biosynthesis of AgNPs, including the silver nitrate solution concentration, the ratio of plant extract to silver nitrate solution, the pH of the reaction mixture, and the incubation period. To determine the best conditions for optimum production, UV–vis spectra were documented to detect variations due to differences in the reaction parameters. The effect of the silver nitrate concentration on AgNP formation using *Pergularia tomentosa* is shown in Figure 2A. A distinctive surface plasmon resonance (SPR) peak detected in the visible region ranging from 400 to 500 nm showed the formation of AgNPs. Interestingly, a similar peak did not appear in the UV–vis spectra of the AgNO_3_ solution or *Pergularia tomentosa*. Additionally, the height of the absorbance peak amplified with increasing concentration of AgNO_3_, demonstrating improved AgNP formation (Figure 2A). The maximum SPR peak intensity was detected at a 5 mM silver nitrate concentration, with a gradual blue shift in λ_max_ from 450 to 436 nm, representing the formation of small AgNPs. Consequently, a 5 mM concentration of AgNO_3_ solution was considered best for further analysis. Shifting the ratios of plant extract to AgNO_3_ in a range of 0.005:1 to 0.06:1 (*v*/*v*) increased the intensity of the SPR peak and AgNP biosynthesis. Compared with other combinations, reacting 5 mM of silver nitrate solution with *Pergularia tomentosa* in a ratio of 0.06:1 narrowed the absorbance maxima at λ_max_ of 438 nm and the maximum synthesis of small monodisperse AgNPs (Figure 2B). A ratio of 0.06:1 (*v*/*v*) between *Pergularia tomentosa* extract and silver nitrate was chosen to optimize other reaction parameters. The pH of the reaction mixture changed in a range of 4–9. The 5 mM AgNO_3_ concentration, the 0.06:1 extract-to-AgNO_3_ ratio, and the color alteration in the reaction mixture also indicated an increased pH. Figure 2C shows the effect of different pH levels on AgNP biosynthesis. When the pH changed from 4 to 6, an SPR peak was absent in the UV–vis spectra, and a widened SPR peak was detected when the pH increased to 7, representing the beginning of AgNP biosynthesis. Moreover, a sharp increase in absorbance peak intensity was documented with a blue shift at a λ_max_ of 452 to 422 nm when the pH increased to 9, representing increased AgNP biosynthesis under alkaline conditions. The effect of incubation time on the biosynthesis of AgNPs was studied by recording UV–vis spectra in time intervals of 15 min, 30 min, 45 min, 1 h, 2 h, 4 h, 8 h, and 24 h from the reaction’s initiation (Figure 2D). The kmax of the absorption maxima differed in a range of 390–440 nm because of the SRP of the AgNPs. The reduction of Ag ions advanced slowly, and the broadening peak after 15 min showed the formation of large poly-dispersed AgNPs. Our results demonstrated a strong SPR peak at a λ_max_ of 418 nm after 24 h of incubation.

### 3.4. Characterization of Silver Nanoparticles

**Figure 3:**
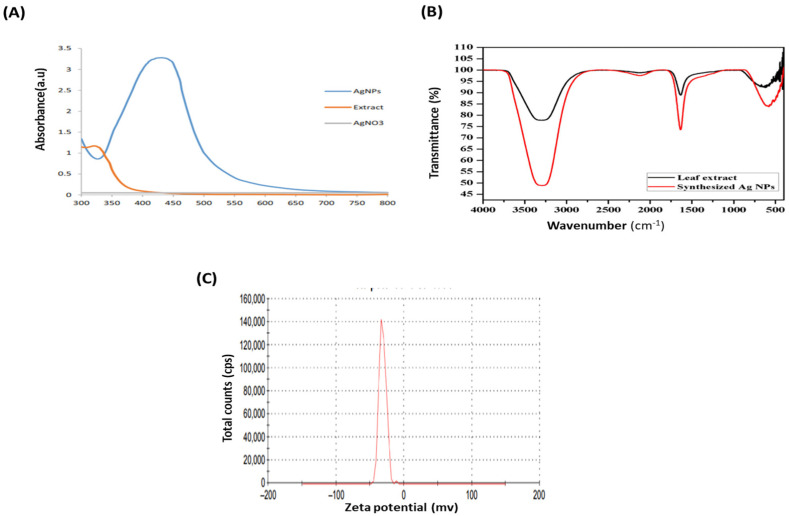
Characterization of PT-AgNPs. (**A**) UV–visible absorption spectra of biofabricated silver nanoparticles, displaying the surface Plasmon resonance peak at 430 nm. (**B**) FTIR spectrum of PT-AgNPs biofabricated using leaf extract of *Pergularia tomentosa.* (**C**) EDX spectrum of PT-AgNPs.

**Figure 4:**
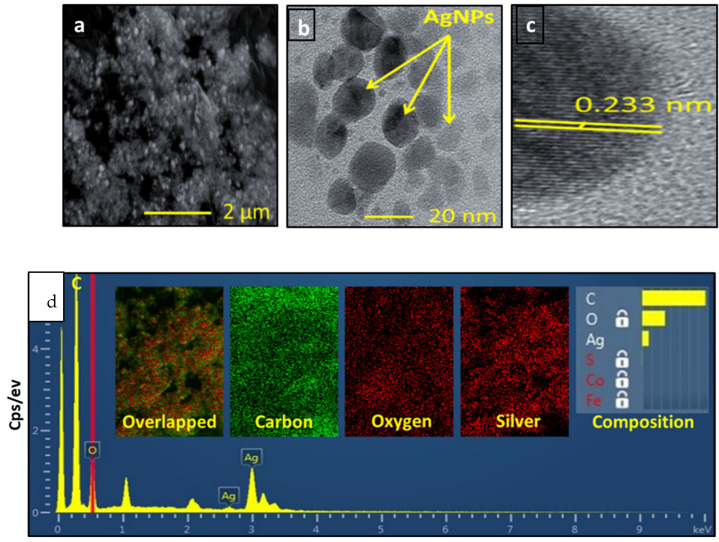
(**a**) Scanning electron microscopy image of PT-AgNPs; (**b**) transmission electron microscopy image of PT-AgPNs; (**c**) high-resolution TEM image of PT-AgNPs displaying lattice fringes; (**d**) energy dispersion X-ray (EDX) images (inset: SEM-derived elemental mapping) of biosynthesis under optimum conditions. Magnification: ×100; scale bar: 50 µM.

**Figure 5:**
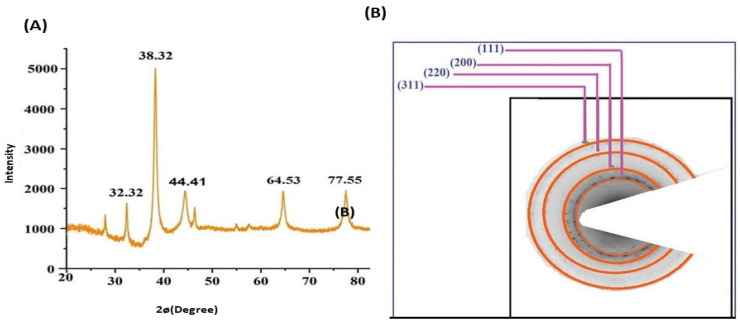
(**A**) XRD pattern and (**B**) SAED pattern of the PT-AgNPs with rings labeled.

**Figure 6:**
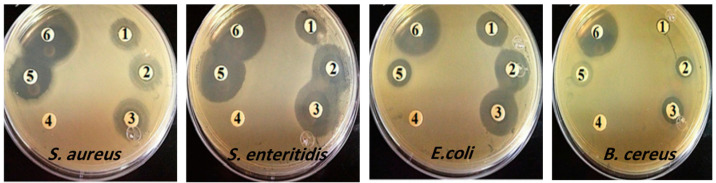
Antibacterial activity of PT-AgNPs, plant extract, and chloramphenicol against *S. aureus*, *S. enteritidis*, *E. coli*, and *B. cereus*. Mueller–Hinton agar plates were swabbed with Mueller–Hinton broth inoculated with different bacterial species and incubated to a turbidity of 0.5 McFarland standard. Filter papers saturated with PT-AgNPs, plant extract, or commercially prepared antibacterial agent disks were placed on the inoculated plates: (1) chloramphenicol, (2) plant extract, (3) ampicillin, (4) DMSO, (5) PT-AgNPs (32 µg/mL), and (6) PT-AgNPs (64 µg/mL).

Based on the results of the previous experiment, we found that 5 mM of AgNO_3_, a 1:0.06 ratio of AgNO_3_ to *Pergularia tomentosa* extract, pH 9.0, and incubating the reaction mixtures for 24 h were the optimum parameters for biosynthesizing silver nanoparticles (PT-AgNPs). Consequently, these PT-AgNPs were characterized using UV–vis spectroscopy by scanning them over a wavelength range of 300–800 nm. This provided a qualitative estimate of the size, shape, and yield accompanied by the agglomeration state of the AgNPs in the suspension. The green biosynthesized AgNPs revealed a distinctive SPR peak at 430 nm, while the positive and negative controls (silver nitrate and *P. tomentosa*, respectively) did not display any (Figure 3A). This color signified the production of AgNPs. The FTIR spectrometry results for the *P. tomentosa* leaf extract and the PT-AgNPs are shown in Figure 3B. The FTIR spectra of plant extract revealed several distinctive peaks at 3165 cm^−1^, corresponding to –OH stretching vibration; 1722 cm^−1^, attributable to –C–H stretching vibration; 1615 cm^−1^, associated with –C=O stretching in the –COOH group of polyphenols; and 1400 cm^−1^ and 1039 cm^−1^ for –C–O stretching vibrations. These distinctive peaks validate the presence of polyphenolic compounds in the plant extract, which could act as a reducing agent in the biosynthesis of PT-AgNPs via AgNO_3_ reduction. The FTIR spectra of the PT-AgNPs displayed distinctive peaks at 3165 cm^−1^, 1615 cm^−1^, and 1400 cm^−1^ for –OH stretching and –C=O and –O–C– stretching vibration, indicating polyphenolic compounds capped on the surfaces of the PT-AgNPs. The peak at 1112 cm^−1^ corresponded to –C–O stretching. The peaks at 617 cm^−1^ and 482 cm^−1^ were attributed to an –O–H bond between the oxygen atom of Ag_2_O and the H atom of the phenolic compound capped on the surface of the PT-AgNPs, indicating the biosynthesis of silver nanoparticles. The surface charges of the PT-AgNPs were evaluated using their zeta potential value. A zeta potential of −32 mV was measured, signifying good stability of the biosynthesized AgNPs (Figure 3). The morphology of the PT-AgNP particles was examined using SEM (Figure 4a). A TEM image revealed that the particles were frequently spherical. The PT-AgNPs were also estimated to have an average diameter of 25.4 nm, and some particles appeared to be aggregated (Figure 4b). Furthermore, the widened portion of a single particle was examined using HRTEM (Figure 4c), displaying a substantially crystalline particle surface with noticeable lattice fringes with 0.233 nm d-spacing. This creates a face-centered cubic (FCC) silver crystal with (111) and (200) planes. A silver nano-metallic signal, rather than silver compounds, was found via EDX examinations (Figure 4d). A sharp peak in the EDX line curve at 2θ = 3.1 keV represented AgNPs in the solution. The obvious color difference in the SEM elemental mapping images supports the presence of PT-AgNPs (Figure 3C). Obviously, other major signals due to carbon (C) and oxygen (O) atoms can also be identified in both the mapping image and the EDX. The X-ray diffraction pattern of the PT-AgNPs is shown in Figure 5A. Distinctive peaks in the XRD diffractogram at values of 38.32, 44.41, 64.53, and 77.55 can be attributed to Ag metal and correspond to (hkl) values of (111), (200), (220), and (311) for planes of silver. These patterns were compared with the diffractogram from JCPDS’s standard powder diffraction card, silver file No. 04–0783. These reflections are inconsistent with a silver metal with face-centered cubic symmetry when compared with the JCPDS file. The high-intensity peak of the FCC materials appears as a (111) reflection, representing the high crystallinity of the nanoparticles. Furthermore, the crystalline nature of the PT-AgNPs was confirmed using a selective area electron diffraction (SAED) pattern, as shown in Figure 5B. The SAED pattern revealed several bright rings with spots in addition to other rings similar to spheres and other irregular shapes. The (111), (200), (220), and (311) Bragg reflection planes were signified by these ring patterns (Figure 5B). The brightest ring and the most intense peak in the SAED and XRD patterns indicate that the crystals were frequently oriented along the (111) Bragg reflection plane. To confirm the morphological characteristics of the PT-AgNPs, transmission electron microscopy was carried out. The average size of the PT-AgNPs was 15 to 50 nm. Figure 6(1–4) shows a micrograph of the PT-AgNPs biosynthesized via green morphology with identical spherical shapes.

### 3.5. Antibacterial Susceptibility

**Figure 7:**
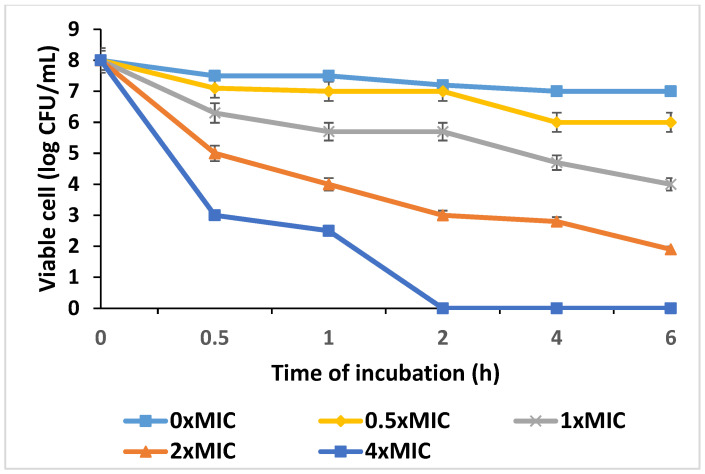
Time–kill curve plots for *S. enteritidis* after exposure to PT-AgNPs.

The results of the antibacterial test of the PT-AgNPs against *E. coli*, *S. enteritidis*, *B. cereus*, and *S. aureus* using the agar disc diffusion technique are shown in Figure 6. Based on the inhibition zone diameter, all tested bacteria were considered resistant to chloramphenicol (with a resistance of less than 28 mm to chloramphenicol). Additionally, *S. enteritidis* was the strain most susceptible to PT-AgNPs. For *S. enteritidis*, PT-AgNPs, plant extract, and chloramphenicol showed mean inhibition halos of 56, 32, and 20 mm, respectively. The Kirby–Bauer assay using CLSI breakpoints revealed that *S. enteritidis* was susceptible to PT-AgNPs, with an MIC of 64 µg/mL (Table 1). In addition, the time–kill assays revealed that the PT-AgNPs killed *S. enteritidis* at 4 × MIC after 2 h (Figure 7).

### 3.6. SEM Examination of PT-AgNP Action Against S. enteritidis

**Figure 8:**
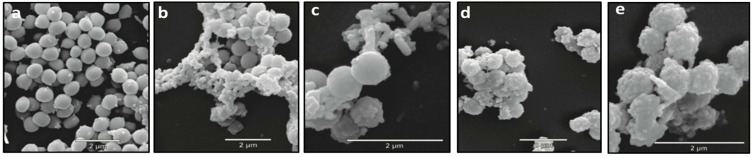
Scanning electron microscope images of PT-AgNP antibacterial activity against *S. enteritides* after 4 h of incubation. (**a**) Negative control (without PT-AgNPs) (25,000×); (**b**) cells treated with chloramphenicol (MIC) (25,000×); (**c**) cells treated with chloramphenicol (MIC) (50,000×); (**d**) cells treated with PT-AgNPs (MIC) (25,000×); (**e**) cells treated with PT-AgNPs (MIC) (50,000×). Different morphological alterations could be detected after the PT-AgNP treatment.

SEM examination showed that there were many untreated smooth *S. enteritidis* cells that had intact surfaces and a constant average size, with distinctive structures that had similar organization, arrangement, and form as was found in the treated cells (Figure 8a). *S. enteritidis* cells treated with chloramphenicol had normal form, arrangement, and organization, although some showed distortions; the images revealed the development of a large amorphous mass resulting from damage to the bacterial cells in the chloramphenicol treatment (Figure 8b,c). The PT-AgNP treatment modified the cell morphology, creating several small bubble protrusions only nanometers in size. The PT-AgNPs also led to several lysed cells and cell debris (Figure 8d,e).

### 3.7. Effect of AgNPs on Membrane Permeability

**Figure 9:**
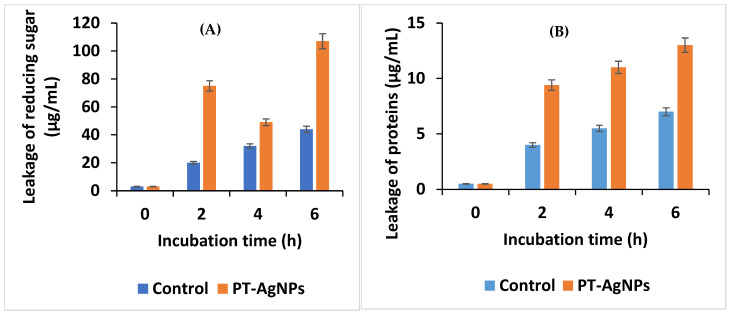
Effect of PT-AgNPs on reducing sugar (**A**) and protein (**B**) leakage from *S. enteritidis*.

The results revealed that the PT-AgNPs could increase the permeability of the cell membrane and consequently increase sugar and protein leakage. The release of sugars and proteins from *S. enteritidis* significantly increased in a time-dependent manner. After 2, 4, and 6 h of incubation, the number of sugars and proteins increased compared with the control (Figure 9A,B). The greatest quantities of sugars and proteins were released in PT-AgNP-treated samples after 6 h (111.58 and 13.8 μg/mL, respectively).

### 3.8. Antioxidant Activity

**Figure 10:**
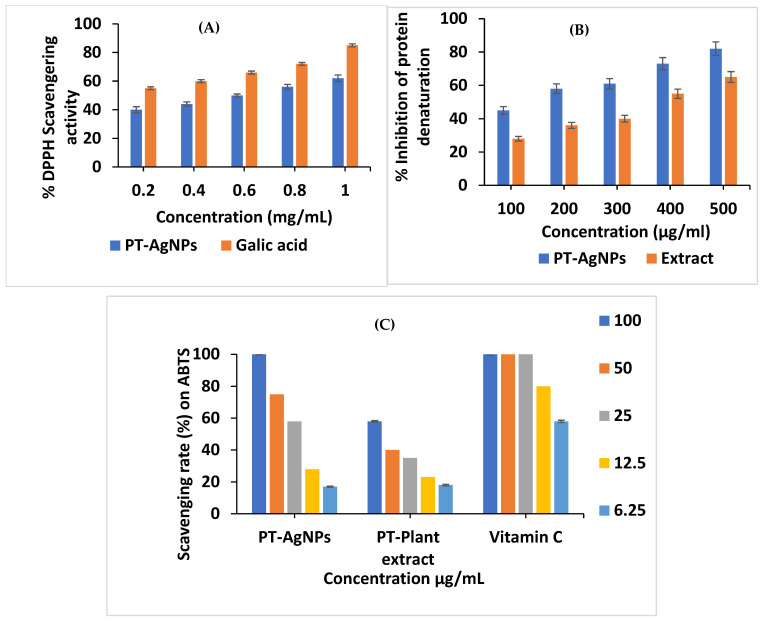
Estimation of antioxidant and anti-inflammatory activities of PT-AgNPs. (**A**) Assessment of DPPH radical scavenging action of different concentrations of PT-AgNPs biosynthesized using *P. tomentosa* extract. (**B**) Anti-inflammatory action of the PT-AgNPs from the *P. tomentosa* leaf extract. (**C**) Scavenging effects of PT-AgNPs on ABTS free radicals.

Using gallic acid as a standard, the DPPH radical scavenging activity of PT-AgNPs of various concentrations (0.2–1.0 mg/mL) was analyzed (Figure 10A) based on a color change from violet to yellow. Gallic acid demonstrated the greatest reducing action, with a scavenging efficiency of 61.79% at 1 mg/mL. The half-maximal inhibitory concentration (IC_50_) value for the PT-AgNPs was 0.69 mg/mL, and the IC_50_ value of gallic acid was 0.13 mg/mL. Colorless ABTS can be oxidized by K_2_S_2_O_4_ to create a stable blue–green ABTS+ free radical, which can then revert to colorless ABTS when combined with antioxidants. Furthermore, ABTS+ exhibits the highest absorption at 734 nm. A comparison of the absorbance change at 734 nm was used to assess how well the tested substances removed ABTS+. Figure 10C and Table 2 show the IC_50_ values of the PT-AgNP-treated samples’ effective components, the positive control (vitamin C (VC)), and their relative scavenging capacities at various doses. Similar to the DPPH tests, the PT-AgNPs proved to be the most potent component, with an IC_50_ value of 22.30 μg/mL. This was significantly higher than those for the other three components, possibly because of the antagonistic action between these elements.

### 3.9. The Isoradiation Method Was Employed to Analyze the Synergistic Effect of PT-AgNPs on ABTS Free Radicals

As previously noted, five combination ratios (0.2, 0.4, 0.6, 0.8, and 1) were examined for the four types of PT-AgNP components that were constructed. For each drug, the ABTS clearance rate at various concentrations was ascertained, and concurrently, the corresponding IC_50_ value at each percentage was determined. Table 3 presents the interaction coefficients (γ).

In contrast to the DPPH test, most of the PT-AgNPs demonstrated a strong effect on scavenging ABTS free radicals and continued to be the most effective. At a mixing ratio of 1, the strongest coefficient (γ) was 0.34. The control had no discernible effects.

### 3.10. Anti-Inflammatory Action

#### 3.10.1. Suppression of Protein Denaturation

Protein denaturation inhibitors are drugs primarily used to block the action of COX enzymes. Consequently, we determined the abilities of the plant extract and biosynthesized PT-AgNPs to suppress protein denaturation. The plant extract and biosynthesized PT-AgNPs exerted a significant anti-denaturation effect in a dose-dependent manner (Figure 10B). At 500 μg/mL, the percentages of protein denaturation suppression were 79% and 59% for the PT-AgNPs and the plant extract, respectively.

#### 3.10.2. Cytotoxicity Studies

**Figure 11:**
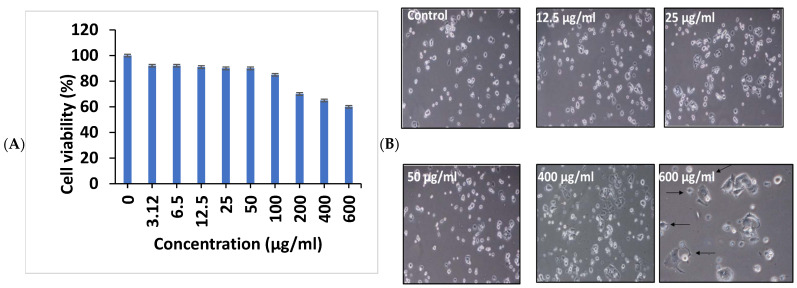
(**A**) Cytotoxic effect of PT-AgNPs on MCF-7 cell viability. The dose-dependent effect of PT-AgNPs on cell viability was measured using an MTT assay after 48 h of treatment. Values are the mean ± SD of three independent experiments. (**B**) The population of MCF-7 cells treated with different concentrations of PT-AgNPs examined under an inverted light microscope in the same spot for 48 h (100×). Cellular shrinkage was observed at 600 µg/mL in the PT-AgNP-treated MCF-7 cells (arrows) (200×).

As an essential step in using PT-AgNPs in vivo, we scanned the cytotoxicity of the PT-AgNPs against MCF-7 as a model human cell line, using a cell viability MTT assay. The PT-AgNPs did not show any signs of cytotoxicity against MCF-7 cells up to the concentration of 120, only affecting MCF-7 cell viability at concentrations of 150 µg/mL (Figure 11A). The population of attached cells in the control—12.5 µg/mL and 25 µg/mL—did not increase after 48 h. For the cells treated with PT-AgNPs at higher concentrations, the cell population increased and the cells detached from the substratum, revealing apoptosis features such as cellular shrinkage at 48 h (Figure 11B).

## 4. Discussion

Biofabricating nanoparticles using plants is a better approach to the economical and large-scale manufacture of NPs because it is eco-friendly [32,33,34,35]. *P. tomentosa* is a medicinal plant characterized by its antioxidant, cytotoxic, and antibacterial potential [36,37,38]. Several biologically active constituents have been extracted from *P. tomentosa*, such as cardenolides [39], glycosides, saponin glycosides, alkaloids, tannins, and flavonoids [40].

This research examined the biosynthesis of AgNPs using a plant-mediated synthetic approach. We are the first to biosynthesize AgNPs using *P. tomentosa* extract, which works as a reductant and capping agent and controls agglomeration. Under the necessary conditions, such as constant stirring and temperature, free silver atoms agglomerated and provided a basis for the formation of colloidal silver nanoparticles. The absorption spectrum of the synthesized nanoparticles was observed at a nanometer scale, which is consistent with the surface plasmon resonance of AgNPs. It is well documented in the literature that the excitation of the surface plasmon vibrations of silver nanoparticles is in the range of 380–450 nm [41]. The FTIR spectrum showed that the band at 3441.01 cm^−1^ matched the vibrations of the OH group of phenolic compounds [41]. The bands at 1120.64 cm^−1^ and 1066.64 cm^−1^ might have been due to the carbonyl vibrations of carboxylic acid from the proteins [42]. The peak at 1408.04 cm^−1^ matched the C=N stretching vibration of aromatic amines [43]. The XRD analysis of AgNPs showed intense peaks ranging from 20 to 80, similar to the Bragg reflection of silver nanocrystals [44]. A TEM image showed that the biosynthesized AgNPs were well dispersed with a hexagonal shape and particle sizes of 5 to 50 nm.

Ali and Abdallah (2020) synthesized AgNPs with similar characteristics using *Calotropis gigantea* extract [45]. EDX revealed that the AgNPs exhibited a typical optical absorption peak at 3 keV because of the surface plasmon resonance [46]. The negative charge value might have been due to the efficient functional components related to the capping agents present in the *P. tomentosa* extract.

The continuous emergence of bacterial species exhibiting resistance to various antimicrobial drugs has encouraged the scientific community to search for novel antimicrobial agents. Therefore, silver, which is generally defined as a wide-spectrum antimicrobial agent, might represent an appropriate choice, particularly given its nanostructure [47]. Numerous studies have shown that green biosynthesized AgNPs have high antimicrobial potential against different groups of microorganisms [48]. Our results revealed the superior antibacterial potential of PT-AgNPs against several tested bacterial species, with MICs ranging from 50 to 100 µg/mL. Numerous studies have described the antibacterial action of AgNPs; however, because of differences in measurement procedures, it is challenging to efficiently compare the results. Several approaches have been proposed to define the suppressive effect of Ag+ on bacteria. For much of history, the silver ion has been recognized as being efficient against an extensive range of microorganisms. The mode of action of silver ion antimicrobial activity is closely related to its interface with thiol (sulfhydryl) groups [49], though other target sites are likely [50]. Amino acids, such as cysteine, and other compounds containing thiol groups, such as sodium thioglycolate, neutralize the effect of silver against bacteria [51]. Conversely, disulfide-bond-containing amino acids, non-sulfur-containing amino acids, and sulfur-containing compounds, such as cystathionine, L-methionine, taurine, and sodium thiosulfate, are incapable of neutralizing the action of silver ions. These findings suggest that the interaction between silver ions and thiol groups in enzymes and proteins plays a major role in silver’s antimicrobial activity, although other cellular constituents, like hydrogen bonding, might also be involved [52]. Silver is also believed to work by binding to major functional groups of enzymes. Silver ions release potassium ions from bacteria; thus, the bacterial plasma or cytoplasmic membrane, which is associated with many important enzymes, is an important target site for silver ions [53].

Velasco-Ramírez et al. (2024) found that disrupting the bacterial membrane structure with AgNPs increased permeability, resulting in the uncontrolled passage of molecules through the cell membrane and finally resulting in cell death [54]. It is believed that Ag+ released from AgNPs can bind with the oxygen, sulfur, and nitrogen of essential biomolecules, thus hindering bacterial growth. An alternative postulated mode of action includes Ag+ inhibiting DNA replication and the proteins essential for synthesizing ATP in bacteria [55]. There are two main factors in this nanoparticle’s effect on living cells. The indirect action of silver can be attributed to the action of Ag+ ions as a soft Lewis acid. The ion has a substantial affinity for elements containing a lone pair of electrons. These elements are nitrogen, sulfur, and other elements that form biomolecules. Having attached to molecules containing these elements, the Ag+ ions interrupt the biochemical processes, thus ceasing the vital activity of cell structures. It is generally believed that the adversarial action of silver ions is due to interactions with thiols and amino groups of proteins, nucleic acids, and cell membranes.

To recognize modifications in the structure of bacterial cells after treatment with silver nanoparticles, we conducted SEM analyses of both the control and AgNP-treated cells. Our results revealed alterations in the AgNP-treated bacterial cells. Additionally, we found that AgNPs could increase membrane permeability and, consequently, sugar and protein leakage. This accords with the findings of Ahmad S. et al. (2024), who observed critical changes and damage in the membrane structure of *E. coli* after AgNP treatment [56]. Similarly, atomic-force microscopic micrographs showed modifications to the ultrastructure and bacterial cell surface after treatment with AgNPs [57]. SEM micrographs showed modifications in the morphology of the bacteria, illustrating the antibacterial mechanisms of biosynthesized AgNPs.

In the current research, the antioxidant action of silver nanoparticles was assessed using DPPH free radical scavenging, with an IC_50_ value of 0.69 mg/mL. The antioxidant activity of AgNPs has been determined and verified in numerous previous studies [58]. Kharat and Mendhulkar (2016) determined the antioxidant action of biosynthesized AgNPs using the DPPH method [59], proposing that AgNPs could be used as free radical scavengers. Similarly, Priya et al. (2016) determined the antioxidant action of AgNPs using *P. pinnata* extract and observed significant free radical scavenging activity [60]. Additionally, Patra and Baek (2016) established their powerful antioxidant potential (IC_50_ of 385.87 µg/mL) [61]. The high antioxidant potential of AgNPs could be due to the simultaneous action of polyphenols in the extract acting as antioxidant components and the AgNPs acting as catalysts [62]. *P. tomentosa* extracts contain several flavonoids, which exert antioxidant activity against DPPH radicals [63]. Therefore, evaluating the antioxidant activity of AgNPs before their use in human applications is important.

Protein denaturation causes inflammation in several conditions, such as rheumatoid arthritis [64]. Inhibiting protein denaturation is the key mode of action in nonsteroidal anti-inflammatory medications, blocking COX enzymes [65]. Consequently, the capacity of the extract and PT-AgNPs to inhibit protein denaturation may contribute to their anti-inflammatory activity. Our results demonstrate that the secondary metabolites of *P. tomentosa* covered the silver nanoparticles. It has been formerly proposed that the secondary metabolites of an extract can reduce acetylcholinesterase activity and induce glutathione S-transferases [66]. In this context, AgNPs biosynthesized using *Cotyledon orbiculata*, curcumin, and *Asparagus racemosus* extracts have been shown to reduce pro-inflammatory cytokine levels, showing that they have anti-inflammatory activity [67]. The higher surface area-to-volume ratio of AgNPs was suggested as one reason for their superior ability to inhibit pro-inflammatory cytokines [68]. Additionally, AgNPs inhibit the production of pro-inflammatory cytokines, decreasing COX-2 gene expression, which promotes anti-inflammatory activity [69]. To the best of the authors’ knowledge, this research is the first to focus on the anti-inflammatory activity of silver nanoparticles that were biosynthesized using *P. tomentosa*.

Our results revealed that using PT-AgNPs is safe in animal cells, suggesting the possible use of PT-AgNPs in vivo in a clinical study. The potential cytotoxicity of AgNPs depends on the routes of administration and the properties or characteristics of the AgNPs, such as their size, shape, and concentration. The cytotoxicity of AgNPs in human cells depends on the nature of the reducing agent [69]. Silver metal and dressings, when used in reasonable quantities, have no negative effects on the human body and have natural antimicrobial activity [70] toward many pathogens such as bacteria [70]. Plants could act as reducing and stabilizing agents to stabilize AgNPs, decreasing their toxicity. Furthermore, the anti-inflammatory activity of *P. tomentosa* extract might reduce the toxicity of biosynthesized AgNPs [71]. GCMS analysis of *Pergularia tomentosa* leaves revealed that they contain 1-(2-butoxyethoxy), cyclohexane propanol, 2-methoxy6-methyl pyrazine, 11,3-dioxane, 2-propenoic acid octyl ester, dihydro methyl jasmonate, 1,2-benzene dicarboxylic acid, and 4-(bromomethyl) cyclohexane-1-ol, highlighting their antibacterial effects [72].

## 5. Conclusions

Recently, nanotechnology has become an important field of research, as it has various uses in healthcare and industry. This study is the first to biosynthesize AgNPs using a *P. tomentosa* extract. The PT-AgNPs demonstrated broad-spectrum antibacterial action against the pathogenic bacteria that were tested. Moreover, significant antioxidant potential and potent anti-inflammatory properties were also observed. The PT-AgNPs showed no indication of cytotoxicity at various MIC values. Nevertheless, additional studies are essential to evaluate these new biological actions (e.g., antifungal and antidiabetic) and their mechanisms.

## Figures and Tables

**Table 1:** MIC values of different antibacterial agents (512–1 µg/mL) against *S. enteritidis*.

Concentration (µg/mL)	Antibacterial Agent			
	DMSO	PT-AgNPs	Plant Extract	Chloramphenicol
	IZD (mm)			
1	_a_0^a^ ± 0.0	_a_0^a^ ± 0.0	_a_0^a^ ± 0.0	_a_0^a^ ± 0.0
2	_a_0^a^ ± 0.0	_b_15^b^ ± 0.7	_b_2^a^ ± 0.7	_a_0^a^ ± 0.7
4	_a_0^a^ ± 0.0	_c_22^c^ ± 0.5	_c_5^b^ ± 0.5	_a_0^a^ ± 0.5
8	_a_0^a^ ± 0.0	_d_33^c^ ± 0.5	_d_8^b^ ± 0.5	_a_2^a^ ± 0.8
16	_a_0^a^ ± 0.0	_d_33^d^ ± 0.5	_e_15^c^ ± 0.5	_b_8^b^ ± 0.0
32	_a_0^a^ ± 0.0	_e_42^d^ ± 0.5	_f_19^c^ ± 0.5	_c_13^b^ ± 0.7
64	_a_0^a^ ± 0.0	_f_56^d^ ± 0.5	_g_23^c^ ± 0.5	_d_16^b^ ± 0.5
128	_a_0^a^ ± 0.0	No growth	_h_28^c^ ± 0.5	_e_20^b^ ± 0.8
256	_a_0^a^ ± 0.0		_i_32^b^ ± 0.5	No growth
512	_a_0^a^ ± 0.0		No growth	

IZD = Inhibition zone diameter (mm). Data are expressed as the mean zone of inhibition in mm followed by SD. The values with different subscript letters in the same column and those with different superscript letters in the same row are significantly different according to ANOVA and Duncan’s multiple range tests.

**Table 2:** IC_50_ value of ABTS radical scavenging from each functional component of the PT-AgNPs.

Components	PT-AgNPs	Control
IC_50_ (μg/mL)	23.33 ± 1.90	5.501 ± 2.06

**Table 3:** Effects of PT-AgNPs on scavenging ABTS free radicals.

Type of Compound	Proportion	The IC_50_ Value of the ABTS Scavenging Effects of the Compounds (μg/mL)		
		Theoretical IC_50_ Value	Measured IC_50_ Value	Γ
PT-AgNPs	0.2	59.33	75.77	1.31
	0.4	42.46	40.90	0.88
	0.6	33.95	21.22	0.69
	0.8	28.90	17.35	0.60
	1	24.95	11.12	0.34
*P. tomentosa* leaf extract	0.2	71.77	124.43	1.20
	0.4	76.65	145.26	1.17
	0.6	84.44	139.20	0.83
	0.8	89.31	213.43	1.11
	1	96.26	326.7	1.09

## Data Availability

Data are contained within the article.

## Supplementary Materials

The following supporting information can be downloaded at: https://www.mdpi.com/article/10.3390/life14121639/s1. Table S1: Phytochemical screening of aqueaus crude extract of P. tomentosa. Table S2: Chemical compounds and their retention time (min) identified in the ethanolic crude extract of P. tomentosa using GC-MS. Figure S1: GC/MS chromatogram of *P. tomentosa* leaf extract.
