# Supplementary material for: Biofabrication of Silver Nanoparticles Using Pergularia tomentosa Extract and Evaluation of Their Antibacterial, Antioxidant, and Cytotoxic Properties
Source: Life (Basel). 2024 Dec 10;14(12):1639. doi: 10.3390/life14121639 (PMC11677515; doi:10.3390/life14121639)
Supplement: Supplementary file 1 [file life-14-01639-s001.zip › life-3252641-supplementary.pdf]

Supplementary Table S1. Phytochemical screening of aqueaus crude extract of *P. tomentosa* .

No	Secondary metabolite	Extract
1	Alkaloids	+++
3	Flavonoids	+++
6	Tannins	++
7	Glycosides	+
8	Saponins	++

Supplementary Table S2. Chemical compounds and their retention time (min) identified in the ethanolic crude extract of *P. tomentosa* using GC-MS.

No.	Compound name	Retention time (min.)	Peak area (%)
1	4-(Bromomethyl) cyclohexane-1-ol	8.95	2.05
2	1,2-Benzene dicarboxylic acid	10.63	7.22
3	Dihydro methyl jasmonate	11.66	7.54
4	2-Propenoic acid octyl ester	13.11	6.21
5	11,3-Dioxane (CAS)	14.35	6.6
6	Tricyclo[5.1.0.0(2,8] octane benzenedicarboxylic acid	14.81	14.3
7	2-Methoxy-6-methyl pyrazine	15.29	13.5
8	Cyclohexanepropanol	15.90	11.2
9	Ethanol,1-(2butoxyethoxy)	17.98	16.7

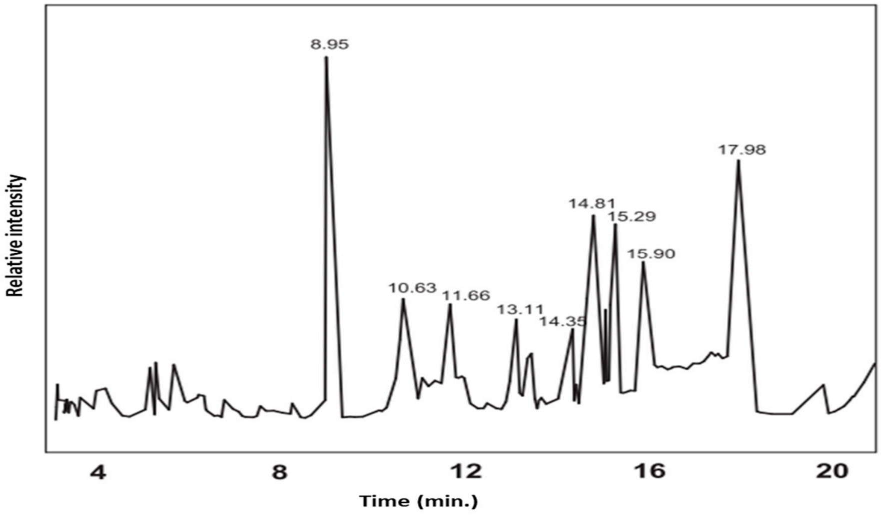

Supplementary Figure S1. GC/MS chromatogram of ***P. tomentosa*** leaf extract
